# Alcohol marketing and drunkenness among students in the Philippines: findings from the nationally representative Global School-based Student Health Survey

**DOI:** 10.1186/1471-2458-13-1159

**Published:** 2013-12-10

**Authors:** Monica H Swahn, Jane B Palmier, Agnes Benegas-Segarra, Fe A Sinson

**Affiliations:** 1 School of Public Health, Georgia State University, P.O. Box 3995, 30302-3995, Atlanta, GA, USA; 2 National Epidemiology Center, Department of Health, Manila, Philippines

**Keywords:** Alcohol, Alcohol marketing, Drunkenness, Philippines, Survey

## Abstract

**Background:**

A largely unaddressed issue in lower income countries and the Philippines, in particular, is the role of alcohol marketing and its potential link to early alcohol use among youth. This study examines the associations between exposures to alcohol marketing and Filipino youths’ drinking prevalence and drunkenness.

**Methods:**

Cross-sectional analyses were used to examine the Global School-based Student Health Survey (GSHS) conducted in Philippines (2011). The self-administered questionnaires were completed by students primarily 13 to 16 years of age (N = 5290). Three statistical models were computed to test the associations between alcohol marketing and alcohol use, while controlling for possible confounding factors.

**Results:**

Alcohol marketing, specifically through providing free alcohol through a company representative, was associated with drunkenness (AOR: 1.84; 95% CI = 1.06–3.21) among youths after controlling for demographic and psychosocial characteristics, peer environment, and risky behaviors. In addition, seeing alcohol ads in newspapers and magazines (AOR: 1.65, 95% CI = 1.05–2.58) and seeing ads at sports events, concerts or fairs (AOR: 1.50, 95% CI = 1.06–2.12) were significantly associated with increased reports of drunkenness.

**Conclusions:**

There are significant associations between alcohol marketing exposure and increased alcohol use and drunkenness among youth in the Philippines. These findings highlight the need to put policies into effect that restrict alcohol marketing practices as an important prevention strategy for reducing alcohol use and its dire consequences among vulnerable youth.

## Background

Alcohol use is the most commonly used psychoactive substance in the world and is one of the leading causes of death and disability [1]. Alcohol abuse causes 3.2% of all deaths worldwide annually and also accounts for 4.0% of the global disease burden each year [2]. Research has shown that alcohol use is associated with alcohol addiction [3], other drug use [4], unintentional injuries [3,5], physical fighting [6], criminal activity [4], suicidal ideation and attempts [7-9], and increased risk of HIV/AIDS [10,11].

In order to address this global public health issue, the World Health Organization (WHO) recently prioritized the global reduction of the harmful use of alcohol [12]. Even with limited data, it is still evident that low and middle-income countries bear a disproportionate public health burden due to increasing alcohol consumption and limited or non-existent prevention policies and programs [1].

Alcohol use among youth is affected by a range of psychosocial and environmental factors. Relatively recent research has increasingly focused on the role of exposure to alcohol marketing broadly defined and its influences on youth drinking in particular. However, such research has been largely limited to high income countries, with a few exceptions [13-17].

Intriguingly, research on the predictors of alcohol use and its adverse outcomes among youth is scarce in the Philippines. Data from the WHO indicates that almost 9% of the Philippines population who are 15 years of age and older (estimated at 86 million) have an alcohol use disorder [18]. In addition, 25% of males and 8.3% of females (15-85+ years) are heavy episodic drinkers [18]. A related and also largely unaddressed issue in the broader Western Pacific region, and the Philippines in particular, is the role of alcohol marketing and its potential link to early alcohol use among youth. Since the 1990s, concern has grown about heavy drinking and alcohol-related harm, and the link with the growth in alcohol marketing that targets young people [19,20]. In the Philippines, new marketing strategies for beer and spirits are now being used to target youth and women, particularly by the large local companies, Asia Brewery and San Miguel Corporation [20]. Although the Philippines has a national legal minimum age for off and on premises sales of alcoholic beverages [18] and a recently enacted drunk driving law [21], there are no restrictions on the marketing of such alcoholic beverages to youth and minors. A high proportion of marketing expenditures are on non-media forms of promotion [20]. Alcohol company sponsorship of sports and cultural events is a major marketing strategy, which is under-researched and rarely addressed by policy makers [20]. Sponsorships at sports events in particular (which attract more young people) provide promotional opportunities that imprint brand names and products on young consumers and potential consumers [20]. Direct marketing includes brand promotions at venues or retail outlets at which drinkers can be approached directly, or creating the brand’s own events at which the public or invited customers attend. New brands and products, such as ready-to-drinks (RTDs) are often launched in this way to give people an opportunity to sample the product [20].

Analysts of the Asian alcohol markets describe RTDs as a starting point for young consumers moving from non-alcoholic beverages to alcoholic drinks. Growth in RTDs is anticipated in the Philippines among women and new young drinkers [22]. Marketing of RTDs in the Philippines began on a small scale but volume sales increased markedly as local companies began to compete with the imported brands led by Diageo Philippines Inc. [22]. There is intense competition between global alcohol companies such as Diageo, Heineken, Carlsberg, Anheuser Busch, SABMiller and Kirin to position themselves to get a share of the emerging markets in the Western Pacific region. Review of recent corporate reports of global alcohol companies shows that the strategy is to target growing countries with high youth populations [20].

In two of the few studies conducted on exposure to alcohol marketing among youth in a low income country, findings demonstrate that alcohol marketing, specifically through the provision of free alcohol to school-attending youth (ages 13–16), is relatively common in Zambia (30%) [23] and among vulnerable service seeking youth in Uganda (27.0%) [24] and that this form of marketing is associated with problem drinking and drunkenness [23,24]. Previous research conducted primarily in North America and Europe shows that exposure to alcohol advertising and ownership of alcohol promotional items has been found to increase the risk of alcohol use among adolescents [14,25]. Moreover, based on extensive research, it is clear that alcohol marketing also influences youths’ attitudes and perceptions about alcohol, which are related to expectancies and intentions to consume alcohol beverages [26,27]. In addition, youth who report liking alcohol advertisements are also more likely to use alcohol [28-30]. More troubling is the issue of the long-lasting effect of alcohol marketing exposure. As an example, research shows that exposure to alcohol advertising in youth predicts youth’s intentions of alcohol consumption up to two years later [31].

The totality of previous research indicate that alcohol marketing to youth is a growing public health concern and that this problem may be exacerbated among youth living in countries with limited alcohol policies and self-regulation by the alcohol industry [32,33]. This may be the case because of the resources available to the alcohol industry to promote their marketing efforts. Alcohol Justice (formerly the Marin Institute) is a group dedicated to respond to the alcohol industry and their marketing practices primarily in the U.S. They report that the alcohol industry spends more than $6 billion each year on marketing its products [34]. Unfortunately, many alcohol marketing practices are aimed directly at youth and those that are outside of the home (e.g., billboards, advertisements at sports events and concerts, buildings, newspapers and magazines, and on the internet) pose particular concerns because parents cannot typically shield their children from those exposures [35]. However, spending on these forms of marketing, labeled “out-of-home advertising” have increased by billions in recent years [35]. Alcohol advertising and marketing of alcohol products clearly increase intent to use as well as actual alcohol use among adolescents [26,27,29,30]. Additionally, recent research shows that youth are more exposed to alcohol marketing than adults and need stronger protections [36]. The increased use of digital media is set to make matters worse. Alcohol marketers are rapidly using social networking for their campaigns, and such media is used more heavily by young people which will likely exacerbate their exposure to alcohol marketing [36].

The purpose of this study is to examine the prevalence of alcohol marketing exposure in a nationally representative sample of youth in Philippines and to examine if there are significant associations between alcohol marketing and drunkenness among Filipino youth. Findings from this study will be important for prevention and intervention efforts that seek to reduce alcohol use and adverse health consequences among youth.

## Methods

The current study is based on the Global School-based Student Health Survey, conducted in Philippines in 2011 among students in grades 1^st^-4^th^ (N = 5290). The GSHS was developed and supported by the World Health Organization in collaboration with the United Nations Children’s Fund, the United Nations Educational, Scientific, and Cultural Organization, the Joint United Nations Programme on HIV/AIDS, and with technical assistance from the Centers for Disease Control and Prevention [37]. The goal of the GSHS is to provide data on health behaviors and relevant risk and protective factors among students across all regions served by the United Nations. Country specific questionnaires, fact sheets, public-use data files, documentation and reports are publicly available from the Centers for Disease Control and Prevention and the World Health Organization [37].

The GSHS is comprised of a self-administered questionnaire, administered to students primarily 11–16 years of age. The survey uses a standardized scientific sample selection process, common school-based methodology, and a combination of core questionnaire modules, core-expanded questions, and country-specific questions. The Philippines GSHS employed a two-stage cluster sample design to produce a representative sample of students in 2nd-4^th^ year levels of Secondary Education or High School. The first-stage sampling frame consisted of all schools containing any of 2nd-4^th^ year levels. Schools were selected with probability proportional to school enrollment size. The second stage of sampling consisted of randomly selecting intact classrooms (using a random start) from each school to participate. All classrooms in each selected school were included in the sampling frame. All students in the sampled class rooms were eligible to participate in the GSHS [37].

Survey procedures were designed to protect students’ privacy by allowing for anonymous and voluntary participation. Students completed the self-administered questionnaire during one class period and recorded their responses directly on computer-scannable questionnaire answer sheet [37].

The current analyses are based on the restricted data file that includes an expanded list of questions. The school response rate was 97%, the student response rate was 84%, and the overall response rate was 82%. A total of 5290 students participated in the Philippine survey [37]. The sample consisted of 2,279 males and 2,986 females, and of the following age groups: 11 years old or younger (n = 20), 12 years old (n = 205), 13 years old (n = 980), 14 years old (n = 1,350) 15 years old (n = 1310) and 16 years old or older (n = 1384).

### Measures

The alcohol marketing factors examined were seeing alcohol advertisements at sports and public events, during sports on TV, on billboards, in newspapers and magazines, seeing actors drink, possessing alcohol brand logo and being offered alcohol from an alcohol company representative. Responses to these questions were dichotomized to indicate any exposure versus none for each of the seven marketing variables (Table 1). The analyses controlled for the following potential confounders (all dichotomized): current alcohol use, bullying victimization, lack of friends, missing school, and illicit drug use (Table 1). Drunkenness, the outcome measure, was assessed through students’ reports of the number of times they had gotten drunk during their lifetime on a 4-item scale ranging from 0 times to 10 or more times. Responses to the outcome measure were dichotomized to reflect none versus at least one episode of drunkenness during lifetime.

**Table 1:** Variable name, description and prevalence of variables examined in the GSHS study of Philippine school students (2011)

**Variable name**	**Variable description**	**N = 5290 Wtd.%**
Current alcohol use	Students who had at least one drink containing alcohol on one or more days during the past 30 days.	23.3%
Drunkenness	Students who drank so much alcohol that they were really drunk one or more times during their life	20.7%
Bullying victimization	Students who were bullied on one or more days in the past 30 days	47.6%
No friends	Students who have no close friends	3.6%
Missed school	Students who missed classes or school without permission on one or more days during the past 30 days	33.8%
Drug use	Students who have ever used drugs such as marijuana, shabu, ecstasy, or rugby	4.9%
Alcohol marketing		
Public ads	Students who went to sports events, fairs, concerts and who most of the time or always saw ads for alcohol	10.0%
Actors	Students who watched actors drinking alcohol on television, videos, or movies.	27.7%
Brand name	Students who watched sports events or other programs on TV during the past 30 days, and who most of the time or always saw alcohol brand names	25.5%
Billboards	Students who have seen a lot of advertisements for alcohol on billboards in the past 30 days.	24.4%
Newspapers/magazines	Students who saw a lot of ads or promotions for alcohol in newspapers or magazines during the past 30 days	16.9%
Own brand logo item	Students who have a t-shirt, pen, backpack, or other item, with an alcohol brand logo on it	14.7%
Offered free alcohol	Students who were ever offered a free drink of alcohol by an alcohol company representative.	10.2%

### Analysis

Logistic regression analyses were computed to determine the associations between alcohol marketing exposures and drunkenness using a 3-step model-building strategy. Model 1 included sex, age, alcohol use in the past 30 days, and usual amount of alcohol use. Model 2 included variables from Model 1 along with bully victimization, lack of friends, missing school, and illicit drug use. Model 3 included variables from Model 1 and Model 2, in addition to factors relating to alcohol marketing, such as seeing alcohol ads at sports events, fairs or concerts, or on TV, seeing actors drink, alcohol advertisements on billboards, and in newspapers and magazines, being offered alcohol from an alcohol company representative and having a brand logo item.

Analyses were conducted with the SAS 9.1 and SUDAAN 10 statistical software packages to accommodate the sampling design, and produce weighted estimates. IRB approval was obtained from the Georgia State University to conduct these analyses.

## Results

The prevalence for each variable examined in this study is outlined in Table 1. Among participants, 23.3% reported current alcohol use and 20.7% reported drunkenness. The bivariate associations between sex, age, and alcohol marketing with current alcohol use, and drunkenness are presented in Table 2. Boys were more likely than girls to report current alcohol use or drunkenness. Youth 16 years of age or older were also more likely to report alcohol use or drunkenness. Exposures to alcohol marketing through seeing actors drinking alcohol on TV, alcohol brand advertising during sports on TV, on billboards, possessing items with an alcohol brand logo and being offered alcohol from an alcohol company representative were associated with increased reports of current alcohol use. All forms of alcohol marketing exposures significantly increased risk for reports of drunkenness, except for newspapers/magazines. Seeing actors drinking on TV significantly increased reports of current alcohol use (OR: 1.48, 95% CI = 1.17–1.87), and drunkenness (OR: 1.25, 95% CI = 1.05–1.49). Seeing alcohol ads on billboards also significantly increased reports of current alcohol use (OR: 1.32, 95% CI = 1.09–1.59), and drunkenness (OR: 1.21, 95% CI = 1.09–1.36). Seeing alcohol ads at sports events, fairs or concerts significantly increased reports of drunkenness (OR: 1.71; 95% CI = 1.27–2.30). In addition, being offered free drinks through an alcohol company representative significantly increased reports of current alcohol use (OR: 2.22, 95% CI = 1.57–3.13), and drunkenness (OR: 2.17, 95% CI = 1.47–3.22). Alcohol marketing through receipt of brand logo items significantly increased reports of current alcohol use (OR: 1.86; 95% CI = 1.53–2.27), and drunkenness (OR: 1.43; 95% CI = 1.23–1.67).

**Table 2:** Bivariate associations between demographic characteristics, alcohol marketing, and drunkenness among participants in the Philippine GSHS Study

**Demographic characteristics**	**Current alcohol use**		**Drunkenness**	
	**%**	**OR (95% CI)**	**%**	**OR (95% CI)**
Sex				
Boys	28.74	**1.86 (1.51–2.28)**	24.84	**1.66 (1.33–2.08)**
Girls	17.85	1.00	16.58	1.00
Age				
<=13	14.30	**0.30 (0.18–0.48)**	9.27	**0.19 (0.13–0.29)**
14	15.53	**0.33 (0.23–0.47)**	13.47	**0.29 (0.22–0.38)**
15	25.69	**0.61 (0.47–0.80)**	23.51	**0.58 (0.46–0.73)**
> = 16	36.01	1.00	34.74	1.00
Alcohol marketing				
Public Ads	34.58	1.34 (0.97–1.84)	36.49	**1.71 (1.27–2.30)**
Actors	29.10	**1.48 (1.17–1.87)**	24.17	**1.25 (1.05–1.49)**
Brand name	29.62	**1.52 (1.26–1.84)**	27.06	**1.53 (1.25–1.88)**
Billboards	26.71	**1.32 (1.09–1.59)**	22.85	**1.21 (1.09–1.36)**
Newspaper/Magazines	25.52	1.19 (0.95–1.50)	23.63	1.26 (0.99–1.60)
Provided free alcohol	37.18	**2.22 (1.57–3.13)**	33.44	**2.17 (1.47–3.22)**
Own gift with brand logo	33.05	**1.86 (1.53–2.27)**	25.46	**1.43 (1.23–1.67)**

OR = Odd Ratios; Significant Odd Ratios are presented in bold face.

Multivariate analyses presented in Table 3, show that current alcohol use was the strongest correlate of drunkenness across the three models computed. In Models 2 and 3, having missed school and other drug use were also associated with increased reports of drunkenness. In Model 3, which examined the potential role of alcohol marketing factors, having received free alcohol from a company representative was significantly associated with drunkenness after controlling for demographic characteristics, personal competencies and peer environment (AOR: 1.84, 95% CI = 1.06–3.21). In addition, seeing alcohol ads in newspapers and magazines (AOR: 1.65, 95% CI = 1.05–2.58) and seeing ads at sports events, concerts or fairs (AOR: 1.50, 95% CI = 1.06–2.12) were significantly associated with increased reports of drunkenness.

**Table 3:** Multivariate logistic regression analyses of the associations between demographic characteristics, alcohol marketing and drunkenness among participants in the Philippine GSHS (2011)

	**Three models predicting drunkenness**		
	**Model 1**	**Model 2**	**Model 3**
	**AOR (95% CI)**	**AOR (95% CI)**	**AOR (95% CI)**
Boys	1.24 (0.95–1.63)	1.09 (0.82–1.46)	1.41 (0.96–2.09)
Girls	1.00	1.00	1.00
<=13 yrs	**0.26 (0.18–0.38)**	**0.25 (0.17–0.37)**	**0.22 (0.13–0.37)**
14 yrs	**0.43 (0.34–0.54)**	**0.46 (0.34–0.62)**	**0.47 (0.33–0.68)**
15 yrs	**0.68 (0.51–0.91)**	**0.68 (0.47–0.98)**	0.76 (0.48–1.20)
> = 16 yrs	1.00	1.00	1.00
Current drinking	**15.60 (11.85–20.55)**	**14.05 (10.62–18.59)**	**11.41 (7.92–16.44)**
Bullying victimization	-	1.04 (0.83–1.29)	0.87 (0.65–1.18)
No friends	-	0.62 (0.34–1.12)	0.82 (0.30–2.26)
Missed school	-	**1.65 (1.38–1.97)**	**1.76 (1.37–2.27)**
Drug use	-	**2.80 (1.59–4.93)**	**2.11 (1.05–4.26)**
Alcohol marketing			
Public Ads	-	-	**1.50 (1.06–2.12)**
Actors	-	-	0.96 (0.75–1.22)
Brand name	-	-	0.98 (0.70–1.38)
Billboards	-	-	0.75 (0.52–1.07)
Newspaper/Magazines	-	-	**1.65 (1.05–2.58)**
Provided free alcohol	-	-	**1.84 (1.06–3.21)**
Own gift with brand logo	-	-	1.19 (0.89–1.59)

AOR = Adjusted Odds Ratios with all variables listed in the column included in the model. Reference groups were those who did not report specific risk behavior or alcohol marketing exposure. Significant Odd Ratios are presented in bold face.

## Discussion

This study examined the prevalence of exposure to alcohol marketing practices among nationally representative school-attending youth in the Philippines, and whether exposure to alcohol marketing is associated with drunkenness. The findings show that there is a high prevalence of exposure to different forms of alcohol marketing strategies. The most commonly reported exposures were seeing alcohol use by actors, seeing alcohol name brands at sport events or on TV and seeing billboards with an alcohol advertisement. However, the form of alcohol marketing that appears particularly troubling is provision of free drinks by alcohol companies directly to youth which was reported by 10% of the students. Moreover, receiving free alcohol from alcohol companies remained associated with drunkenness in multivariate analyses indicating that it appears to be a relatively robust risk factor for alcohol misuse. This issue regarding providing free drinks to youth has been observed in other countries [23] and warrants attention by researchers and policy makers.

It is clear from previous research that direct marketing of alcohol products increases alcohol use and problems among youths and those findings are corroborated by the findings in the current study. The results of this study indicate a high prevalence of current alcohol use (23.2%) and drunkenness (20.7%) among the school-attending youth in the Philippines., in addition to a very strong association between current drinking and drunkenness. Our findings are also supported by research in the U.S. that shows that distributing alcohol merchandise to youth predicts their alcohol use [38]. In this study, nearly 15% of youth reported owning an item with an alcohol logo on it. However, owning an item with an alcohol brand logo on it was not associated with drunkenness.

The findings of this study show that alcohol marketing strategies in the Philippines through providing free alcohol to youth and through print and television media appear to reach a relatively large population of youth. These findings regarding the association between provision of free alcohol to youth and self-reports of drunkenness, mirror those conducted in Sub-Saharan Africa [23]. These alcohol marketing practices, aimed directly to children, have been banned in other countries and have important policy implications for countries where such bans do not exist. The implications of the empirical findings from the current study clearly indicate that stricter policies to prevent underage alcohol advertisements are needed. Such measures need to be urgently considered and applied given the frequency and levels of exposure to alcohol marketing, in particular, the free distribution of alcohol to youths, as observed in the current study.

The timing is critical for new policy initiatives and prevention strategies aimed to reduce alcohol use among adults and youth [39]. Recent reports by the alcohol industry indicate that they will produce and sell cheaper alcohol to Asia and African markets in order to increase its consumer market [20]. The goal of targeting low-income consumers and creating affordable brews will be achieved through using local ingredients, and also utilizing inexpensive individual-sized packaging to make purchasing alcohol more affordable [20]. Previous research clearly highlights that affordability of alcohol is strongly linked to alcohol use [40], and that these new industry strategies are likely to have a negative impact on alcohol use and alcohol-related adverse outcomes among youth in the Philippines and the Western Pacific region.

Policy and intervention suggestions for agencies provided by the World Health Organization to counteract alcohol marketing and reduce harmful effects of alcohol use include regulating alcohol marketing content and the volume of marketing, regulating marketing in media and sponsorship activities of alcohol industry, restricting or banning alcohol promotions targeting young people, regulating alcohol marketing techniques like social media, developing effective surveillance systems to monitor alcohol marketing, and enforcing marketing restrictions [12]. More research is necessary regarding the exposure of youth to alcohol advertising and levels of consumption to gain formative information needed to counteract these marketing influences and inform policy makers to support and implement such strategies.

The WHO has taken an important leadership role related to underage drinking prevention in their Report on the Seventh Meeting of the Regional Advisory Panel on Impacts of Drug Abuse: Technical consultation on the global strategy to reduce the harmful use of alcohol [12]. Per the Report, mandatory as well as voluntary regulations of marketing of alcohol products need to be considered and included in a comprehensive strategy to reduce the harmful use of alcohol. The Report also underscores that these measures need to be urgently considered and applied given the frequency and levels of exposure to alcohol marketing, in particular, the free distribution of alcohol to youths, as observed in the current study.

Although regulation of alcohol marketing to youth has been scarce in low and middle income countries, some progress is currently underway to enact policy in this area. [39]. In Zambia, bans have recently been implemented against the manufacturing and sale of strong liquor individual- sized sachets sold at very low prices, often in unlicensed bars and to minors [41]. Moreover, a recent commentary recommends that there should be a total ban on alcohol advertising in South Africa [42,43]. Additionally, the South African Minister for Social Development, Bathabile Dlamini, has stressed the need for restricting alcohol advertising in the country, in order to reduce the burden of social and health consequences of binge drinking among South Africans [44]. Researchers who have modeled the effectiveness of a ban on alcohol advertising on youth drinking in the U.S. found that among interventions shown to be successful in reducing youthful drinking prevalence, advertising bans appear to have the greatest potential for premature mortality reduction [45]. Another study performed an econometric analysis using data from the National Longitudinal Survey of Youth and estimated that a 28% reduction in alcohol advertising would reduce adolescent monthly alcohol use from 25% to between 24% and 21%, and would reduce adolescent binge drinking from 12% to between 11% and 8% [46]. This study also concluded that a total ban on all forms of alcohol marketing would results in further decreases in alcohol use and binge drinking among youth [47].

These same economists performed a study of 20 countries over 26 years and showed that a ban on all alcohol advertising could reduce underage monthly alcohol participation by about 24% (almost as much as a 100% increase in alcohol prices) and would reduce binge drinking by about 42% [47].

There are several limitations that should be considered when interpreting the findings of this study. First, the study is based on self-reported data of students in the Philippines. Accordingly, the findings may not be generalized to other populations or to youth who are no longer in school. Second, while our findings show statistically significant associations between marketing practices, other correlates, and drunkenness, more specific temporal ordering cannot be determined, nor can causality be inferred. Finally, the study did not include specific measures of other marketing strategies and educational experiences or other factors that may influence or confound the associations observed between alcohol marketing and drunkenness.

## Conclusions

This study demonstrated that there are significant exposure to alcohol marketing and that this exposure is associated with alcohol use and drunkenness among school-attending youth in the Philippines. These findings highlight the need for leaders to prioritize implementation of policies that limit alcohol exposure and that restrict alcohol marketing practices as important prevention strategies for reducing alcohol use and its adverse health consequences among youth in the Philippines.

## Competing interests

The authors declare that they have no competing interests.

## Authors’ contributions

MHS conceptualized the study, guided the analyses and drafted sections of the manuscript. JBP conducted the literature review and drafted sections of the manuscript. ABS led the acquisition of the data and provided contextual information. FAS led the acquisition of the data, and interpreted and reviewed analyses. All authors reviewed multiple versions of the manuscript and read and approved the final manuscript.

## Pre-publication history

The pre-publication history for this paper can be accessed here:

http://www.biomedcentral.com/1471-2458/13/1159/prepub
